# Augmenter of liver regeneration protects the kidney against ischemia-reperfusion injury by inhibiting necroptosis

**DOI:** 10.1080/21655979.2022.2037248

**Published:** 2022-02-14

**Authors:** Yue-Juan Liao, Yi-Xin Ma, Li-Li Huang, Zheng Zhang, Fang-Yan Tan, Li-Li Deng, Dan Cao, Xu-Jia Zeng, Gui-Quan Yu, Xiao-Hui Liao

**Affiliations:** a Department of Nephrology, The Second Affiliated Hospital, Chongqing Medical University, Chongqing, China; b Department of Cell Biology and Genetics, Chongqing Medical University, Chongqing, China; c Department of Nephrology, Chongqing Sanbo Changan Hospital, Chongqing, China; d Department of Nephrology, The Fifth Hospital of Chongqing, Chongqing, China

**Keywords:** Augmenter of liver regeneration, kidney, hypoxia reoxygenation, ischemia-reperfusion, necroptosis

## Abstract

Necroptosis plays an important role in the pathogenesis of acute kidney injury (AKI), and necroptosis-related interventions may therefore be an important measure for the treatment of AKI. Our previous study has shown that augmenter of liver regeneration (ALR) inhibits renal tubular epithelial cell apoptosis and regulates autophagy; however, the influence of ALR on necroptosis remains unclear. In this study, we investigated the effect of ALR on necroptosis caused by ischemia-reperfusion and the underlying mechanism. In vivo experiments indicated that kidney-specific knockout of ALR aggravated the renal dysfunction and pathological damage induced by ischemia-reperfusion. Simultaneously, the expression of renal necroptosis-associated protein receptor-interacting protein 1 (RIP1), receptor-interacting protein 3 (RIP3), and mixed-lineage kinase domain-like protein (MLKL) significantly increased. In vitro experiments indicated that overexpression of ALR decreased the expression of hypoxia-reoxygenation-induced kidney injury molecules, the inflammation-associated factor tumor necrosis factor-alpha (TNF-α), and monocyte chemotactic protein. Additionally, the expression of RIP1, RIP3, and MLKL, which are elevated after hypoxia and reoxygenation, was also inhibited by ALR overexpression. Both in vivo and in vitro results indicated that ALR has a protective effect against acute kidney injury caused by ischemia-reperfusion, and the RIP1/RIP3/MLKL pathway should be further verified as a probable necroptosis regulating mechanism.

## Graphical abstract

**Figure:**
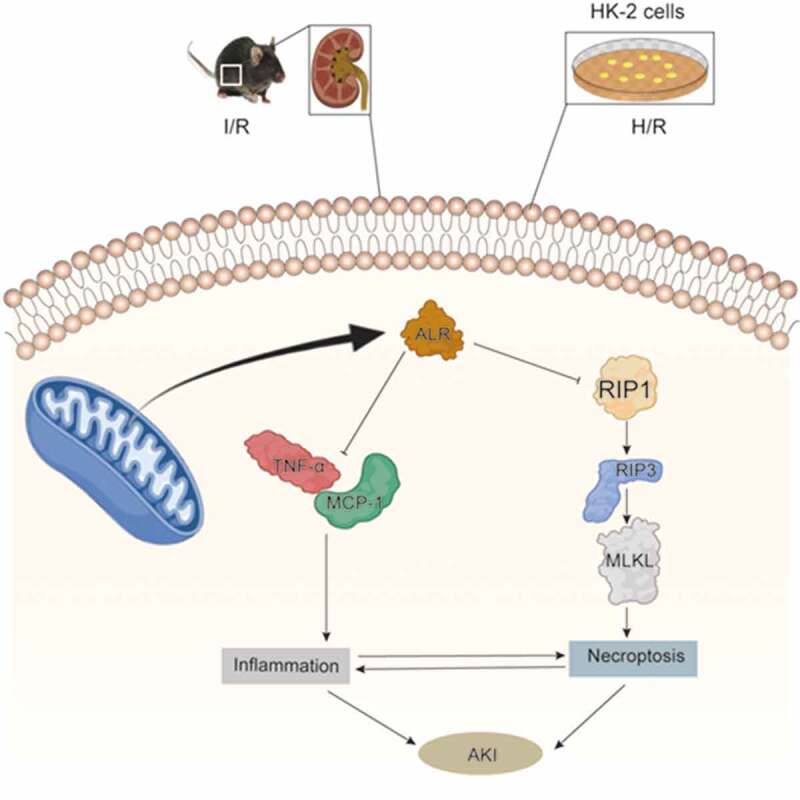

## 1 Introduction

Acute kidney injury (AKI) is a common clinical disease characterized by a rapid decline in renal function within a short time, accompanied by the accumulation of metabolic waste and toxins, which cause a series of clinical complications and can lead to the failure of other organs [1]. Current research has confirmed that AKI can also potentially lead to chronic kidney disease and end-stage renal disease [2]. AKI has now become a global public health problem. Ischemia-reperfusion (IR) is the main clinical cause of AKI. The typical pathological features of AKI are renal tubular injury, inflammation, and vascular dysfunction [3], which eventually lead to acute tubular epithelial cell injury. Previous studies have indicated that ALR is expressed mainly in renal tubules, and ultimately protects renal function by regulating autophagy via the AMP-activated protein kinase (AMPK)/mammalian target of rapamycin (mTOR) pathway, mediating mitophagy via phosphatase and tensin homolog (PTEN)-induced putative kinase 1(PINK1)/Parkin and ferroptosis via glutathione (GSH)/glutathione peroxidase (GPx) system [4–8]. Current research suggests that 15kDa ALR has proliferative and anti-apoptotic effects, and 23kDa ALR plays an important role in inhibiting oxidative stress. AKI-induced epithelial cell damage mainly includes cell apoptosis and necrosis. However, the curative effects of interventions targeting apoptosis or necrosis have been limited [9], thus suggesting that the pathogenesis of AKI has not been fully elucidated. Therefore, the pathogenesis of AKI must be further explored.

Necroptosis, a new form of programmed cell death discovered in recent years, has characteristics of both necrosis and apoptosis. Like apoptosis, necroptosis is regulated by specific molecules; its morphological characteristics in common with necrosis include cell swelling, plasma membrane permeability, and loss of cell contents [10]. Necroptosis may lead to extensive tissue damage in areas including the nervous system, cardiomyocytes, nasopharyngeal carcinoma cells, and the intestinal tract [11–14]. Current research suggests that necroptosis is mediated by the necroptosis complex, a signaling complex including receptor-interacting protein 1 (RIP1) and receptor-interacting protein 3 (RIP3) and mixed lineage kinase-like domains (MLKL) [15]. Caspase-8 is a multifunctional molecule involved in regulating cell death. Activated caspase-8 cleaves RIP1 and RIP3, then induces cell apoptosis. When caspase-8 activity is absent, RIP1 and RIP3 are not cleaved and together cause necroptosis through the combination of receptor-interacting protein (RIP) homotypic interaction motif (RHIM) [16,17]. Some studies have shown that the RIP1 inhibitor Necrostatin-1 (Nec-1) or knockout of RIP3 in mice to block necroptosis markedly alleviates the kidney damage caused by AKI [18,19]. Necroptosis brings about cell death and the development of inflammation, and elevated inflammatory factors further aggravate necroptosis; finally, positive feedback promotes the progression of AKI. Hence, inhibiting necroptosis decreases the production of inflammatory factors and is beneficial for the kidney [20,21].

Augmenter of liver regeneration (ALR) was first isolated from the livers of weanling rats. Because it promotes hepatocyte proliferation, ALR is also called a liver stimulating factor [22]. Previous studies on ALR have focused on the liver, although ALR is a widely distributed multifunctional factor that is also highly expressed in the kidney [22,23]. ALR exists primarily in three forms of 15-kDa, 21-kDa, and 23-kDa. However, few studies have been conducted on 21-kDa ALR in the kidney, and its main function remains unclear. Many studies have examined 15-kDa and 23-kDa ALR in the kidney. The 15-kDa form is mainly distributed in the nucleus and can be secreted into the extracellular environment, and the 23-kDa form is mainly located in the intermembrane space in mitochondria (ISM) [24]. A recent study has found that ALR has anti-apoptotic and anti-oxidative stress effects in addition to stimulating liver cell regeneration [25]. Our study has shown that the expression of ALR in the AKI model is significantly elevated, and exogenous injection of ALR has a protective effect against AKI [26]. Moreover, the overexpression of the ALR gene in an IR in vitro model decreases mitochondrial damage by inhibiting fission and promoting the fusion of the mitochondrial inner membrane [27]. In addition, we found that knocking out ALR in the IR *in vitro* model regulates autophagy and ferroptosis [4,6]. However, to date, no study has reported the regulatory effects and mechanisms of ALR on necroptosis in AKI.

We hypothesized that ALR could alleviate ischemia-reperfusion-induced acute kidney injury by regulating necroptosis. We established an ischemia-reperfusion model through in vivo and in vitro experiments to confirm that ALR can regulate necroptosis through the RIP1/RIP3/MLKL pathway, thereby alleviating acute kidney injury.

## 2 Materials and methods

### 2.1 Mouse ischemia-reperfusion model

Specefic pathogen Free (SPF) grade C57 background kidney-specific knockout 23-kDa ALR mice (genotype ALR^flox/flox^-Ggt1-Cre, referred to as knockout (KO) mice) and control mice (genotype ALR^flox/flox^, referred to as control (CON) mice) were purchased from Saiye Biotechnology Co. Ltd., (Jiangsu, China) and raised in the Animal Experiment Center of Chongqing Medical University (Chongqing, China). The mouse pups were genotyped by polymerase chain reaction (PCR). Briefly, a 0.5 mm tail biopsy was cut, and the deoxyribonucleic acid (DNA) was extracted with a commercial kit (Thermo Fisher Scientific, USA). Primers for Neo-del PCR were as follows: (annealing temperature 62.0℃; Taq for P222-C2) F2: 5′-GCTCAAGCCAATCTCTCAGCTT-3′ and R2: 5′-ATCTAGAGTTTCTAGGTAGGGCCTG-3′. Homozygotes showed one 450 bp band, whereas heterozygotes showed two bands at 450 bp and 327 bp. The wildtype allele showed a 327 bp band. Primers for loxP PCR were as follows: (annealing temperature 62.0℃; Taq for P222-C2) F1: 5′-GTTTGGAGCACGGGAAGCGACCG-3′ and R1: 5′-GTTGACAGCCTACTAAATAACTTCG-3’; targeted: 305 bp. Primers for Ggt1-Cre PCR were as follows: (annealing temperature 62.0℃; Taq for P222-C2) F3: 5′-CATCACATCAGGCACCCCAGAA-3′ and R3: 5′-GAACATCTTCAGGTTCTGCGGGA-3’; Cre amplicon: ~420 bp.

Animal experiments in this study were approved by the Ethics Committee of Chongqing Medical University. Briefly, the light/dark cycle for mouse rearing was 12 hours(h)/12 h, and standard water and diet were provided. When the mice reached 8–12 weeks and weighed 22–25 g, the CON mice were randomly divided into CON group and CON+IR group, and the KO mice were randomly divided into KO group and KO+IR group, with 6–8 mice per group. For establishing the ischemia-reperfusion model, mice were anesthetized with sodium pentobarbital intraperitoneally at a dose of 60 mg/kg. Then the back hair was removed, and the mouse body temperature was maintained at 36.5 to 36.7°C on a temperature-regulated heating pad. After iodophor smearing, a small 1 cm incision was made on both sides of the back spine, the skin, and subcutaneous tissues were carefully separated to expose the kidney. A sterile blood vessel clip was used to clamp the renal pedicle. The kidney and incision were kept moist, and the color change in the kidney was observed during the operation [28]. After 25 mins, the vascular clip was removed, and the kidney was monitored for color recovery. If the color of the kidney recovered, the wound was intermittently sutured with sterile sutures. The mice were sacrificed if the color of the kidney did not recover. Finally, the mice were sacrificed 24 h after reperfusion, and blood samples and kidney tissue were collected for further analysis. In CON group and KO group, the renal pedicle was scarcely separated by blunt dissection and followed by the same treatment as in the IR groups.

### 2.2 Determination of blood creatinine and urea nitrogen

Blood samples were collected retro-orbitally from the sacrificed mice and allowed to stand for 6 h at 4°C. After the serum was separated, the blood samples were centrifuged at 900 × g for 10 mins. The upper layer serum was aspirated and transferred into a new eppendorf (EP) tube [29]. Serum creatinine (Scr; cat. no. S03076) and blood urea nitrogen (BUN; cat. no. S03036) were assessed with reagent kits, according to the manufacturer’s instructions (Nanjing Jiancheng Bioengineering Institute).

### 2.3 Renal histology and immunohistochemistry

Mouse kidney tissue was fixed with 4% paraformaldehyde and embedded in paraffin. Paraffin sections (4 μm) were used for hematoxylin and eosin (HE) staining and periodic acid-Schiff (PAS) staining [7,29]. The renal tubular interstitial damage was scored by determining the percentage of tubules showing renal tubular necrosis. In brief, ten visual fields were randomly selected to observe the renal tubular epithelial cell damage at 400× magnification. Scores of 0–5 points represent damage ranges of 0%, < 11%, 11%–25%, 26%–45%, 46%–75% and > 75%, respectively. For semi-quantitative analyses on the immunohistochemical (IHC)-processed kidney specimens, images were collected and analyzed using open-source software Image Pro Plus software (Media Cybernetics, USA)

### 2.4 Cell culture

Human kidney proximal tubular (HK-2) cells were purchased from the American Type Culture Collection (Rockville, MD, USA) and cultured in Roswell Park Memorial Institute 1640 medium (Gibco, Carlsbad, CA, USA) supplemented with 10% fetal bovine serum (Gibco) and 1% penicillin/streptomycin (Invitrogen, Carlsbad, CA, USA) in a humidified incubator under an atmosphere of 5% CO_2_ at 37°C [4].

### 2.5 Cell transfection

HK-2 cells were seeded in six‐well plates and grown to 70–80% confluence before transfection. Lentiviral vectors were used for cell transfection. Lentivirus particles containing 23-kDa ALR (LV-ALR GV358; GFER (43,708–1); Shanghai Gene Chemistry, China) and lentivirus particle vector (LV-Vector; CON238[UBI-MCS-3flag-SV40-EGFP-Ires-puromycin]) were transfected into HK-2 cells for 8–16 h according to the manufacturer’s instructions. At 72 h after transfection, the cells transfected with the indicated lentivirus vectors were grown in complete medium containing puromycin (3 μg/ml) for selection for 2 weeks. At 72 h after transduction, transduced cells were observed under a fluorescence microscope to verify GFP fluorescence, and cells with an infection efficiency of at least 80% were selected for subsequent experiments [30]. The following primer sequences were used: GFER (43,708–1)-P1: 5′-GAGGATCCCCGGGTACCGGTCGCCACCATGGCGGCGCCCGGCGAGC-3′; GFER(43,708–1)-P2: 5′-TCCTTGTAGTCCATACCGTCACAGGAGCCATCCTTCCAGCC-3′.

### 2.6 HR treatment *in* *vitro*

To induce hypoxia and reoxygenation (HR) in vitro, we placed HK-2 cells in D-glucose free and serum-free Dulbecco’s modified Eagle’s medium (DMEM) for 12 h in advance and incubated the cells to synchronize cell growth. Then, the cells were incubated for 6 hours in a three-gas incubator under hypoxic conditions (94% N_2_, 5% CO_2_ and 1% O_2_ at 37°C). After 6 hours, the cells were transferred to room temperature and cultured in Roswell Park Memorial Institute 1640 complete medium at 37°C and 5% CO_2_ for the specified time points (3, 6, 12 or 24 hours) [30]. If necessary, Nec-1 (50 μmol/L) was administered 12 hours before HR treatment. Cells were collected at the designated time points for subsequent experiments.

### 2.7 Transmission electron microscopy (TEM) examination

The postoperative kidney tissues were immersed in glutaraldehyde, fixed with 1% osmium tetroxide, dehydrated with a series of ethanol gradient solutions for 15 mins, embedded in epoxy resin, sliced into 60 nm serial sections, stained with 2% uranyl acetate and 0.04% lead citrate and finally observed with a transmission electron microscope (TEM, Hitachi, Tokyo, Japan) [29].

HK-2 cells were harvested immediately after the planned treatments. After dehydration with a gradient series of ethanol solutions for 15 mins, HK-2 cells in 10 cm dishes were fixed in 2.5% glutaraldehyde at room temperature for 1 hour, followed by further fixation with 1% OsO_4_. The samples were then embedded in epoxy resin, sliced into serial sections of 60 nm, stained with 2% uranyl acetate and lead citrate and observed using TEM [4].

### 2.8 Evaluation of cell viability

A cell counting kit-8 (CCK-8; Dojindo, Japan) was used to detect cell viability. According to the manufacturer’s instructions, each group of cells was seeded in 96-well plates at a density of 1 × 10 ^4^ cells per well and observed at 0, 24, 48 and 72 hours [4]. A microplate reader (Molecular Devices, LLC, Sunnyvale, CA, USA) was used to measure the absorbance at wavelength of 450 nm. The experiment was repeated three times under the same conditions.

### 2.9 Western blotting

Protein was extracted from mouse kidney tissue or cells with radioimmunoprecipitation assay (RIPA) lysis buffer, and the protein concentrations were detected with a BCA protein concentration determination kit (SolarBio, Beijing, China). An equivalent amount of protein (40 µg) was separated with 10%–15% SDS-PAGE (US Bio-RAD) and transferred to polyvinylidene fluoride membranes with a constant current of 260 mA. The membranes were blocked with skimmed milk powder for 1 hour at room temperature, and specific primary antibodies were added and incubated with shaking overnight at 4°C. After being washed with Tween 20 Tris buffered saline three times, the membrane and peroxidase-conjugated secondary antibody were incubated for 1 h at room temperature. Finally, the membrane was washed three times, and immunoreactive bands were detected with Immobilon Western substrate and a Chemi Doc Imaging System (Bio‑Rad Laboratories, Inc.) and quantitatively analyzed in triplicate by normalization of the band intensities to those of the controls in films scanned with ImageJ v1.8.0 software (National Institutes of Health) [27].

The following antibodies were used: DYKDDDDK Tag (D6W5B) rabbit mAb (1:1,000; Cell Signaling Technology), MLKL (D2I6N) rabbit mAb (1:1,000; Cell Signaling Technology), RIP (D94C12) rabbit mAb (1:1,000; Cell Signaling Technology), RIP3 (E1Z1D) rabbit mAb (1:1,000; Cell Signaling Technology), RIP3 (D4G2A) rabbit mAb (1:1,000; Cell Signaling Technology), MLKL (ab243142) (1:1,000; Abcam), MCP1 (ab151538) (1:5,000; Abcam), TNF alpha (ab215188) (1:1,000; Abcam), KIM-1 (219,211) (1 µg/ml, Novusbio), ALR (1:500; Santa Cruz Biotechnology, Santa Cruz, CA, USA) and GAPDH (D16 H11) rabbit mAb (1:1,000; Cell Signaling Technology). Horseradish peroxidase-conjugated goat anti-rabbit and goat anti-mouse were used as secondary antibodies (1:10,000; Cell Signaling Technology).

### 2.10 Real‑time polymerase chain reaction (RT-PCR)

Total ribonucleic acid (RNA) was extracted from HK-2 cells with a total RNA extraction kit (TaKaRa, Japan). The PrimeScript™ II Reverse Transcriptase Kit (TaKaRa) was used to reverse transcribe total RNA into cDNA for analysis of mRNA levels. Real-time PCR was performed with SYBR Premix (TaKaRa). For quantitative analysis, a CFX Connect Real-Time system was used to amplify the genes with SYBR Premix, and glyceraldehyde 3-phosphate dehydrogenase (GAPDH) served as a positive control (Bio-Rad). The CFX Manager primer sequences were as follows: ALR-F 5′-GTG AGG AGT GTG CTG AAG ACC-3′; ALR-R 5′-TGA GCA GTC GAA GTC AGG CTTG-3′; GAPDH-F 5′-GTCTTCCCCTCCATCGTG-3′ and GAPDH-R 5′- AGGGTGAGGATGCCTCTCTT-3′. All experiments were performed in at least triplicate, with three independent replicates for each group. The messenger RNA (mRNA) levels were normalized to GAPDH mRNA. To quantify gene expression, the 2^−ΔΔCt^ relative quantification method was used [6].

### 2.11 Statistical analysis

Statistical analysis was performed in GraphPad Prism 7.0 (GraphPad Software, Inc.). The data are expressed as mean ± standard deviation of three independent replicates. One-way ANOVA and subsequently Tukey’s post hoc test were used to compare the differences between multiple groups. P < 0.05 was considered to indicate a statistically significant difference.

## 3 Results

This study investigated the effect and underlying mechanism of ALR on necroptosis in IR-induced acute kidney injury. We found that kidney-specific knockdown of ALR aggravated IR-induced acute kidney injury with markedly elevated necroptosis-related proteins. In addition, we transfected HK-2 cells with lentivirus in vitro to establish a cell model of HR-induced AKI, and found that overexpression of ALR can inhibit inflammatory burst and regulate necroptosis. The underlying mechanism may be the inhibition of RIP1/RIP3 /MLKL pathway.

### 3.1 The effect of kidney-specific ALR knockout on renal function and pathological damage in AKI mice

**Figure 1.**
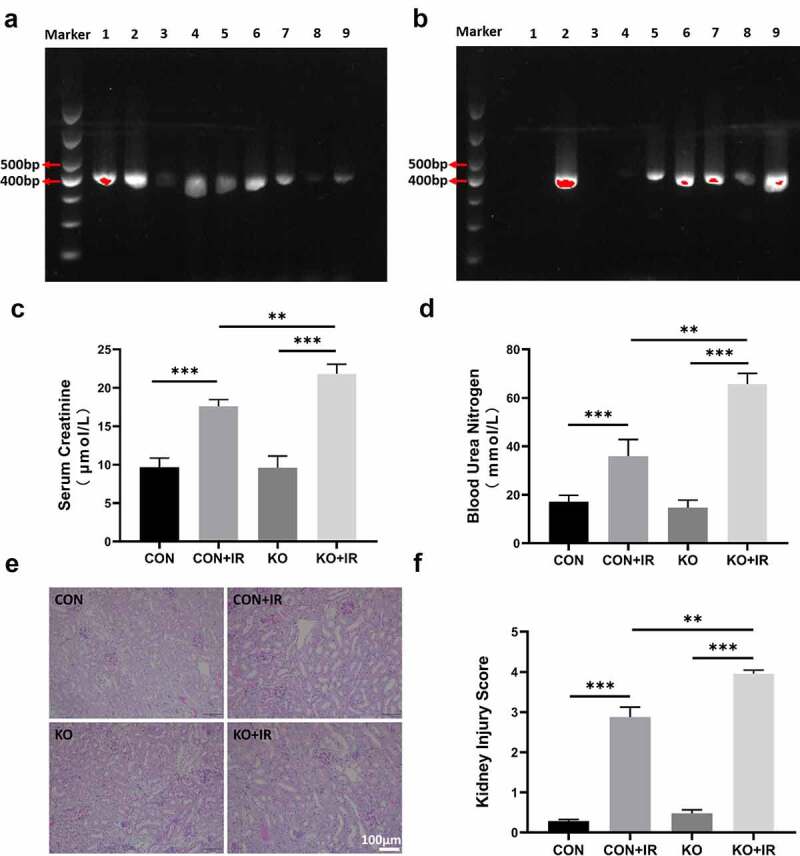
The effects of kidney-specific ALR knockout on renal function and pathological damage in AKI mice. (a, b) Genotype identification of kidney-specific ALR knockout mice. (c) Determination of blood creatinine concentration. (d) Determination of blood urea nitrogen concentration. Representative images (e) and scores (f) of PAS-stained kidney sections. scale bar:100 µm, n = 6–8 mice per group, the bar graph represents the mean ± standard deviation, *P < 0.05, **P < 0.01, ***P < 0.001.

**Figure 2.**
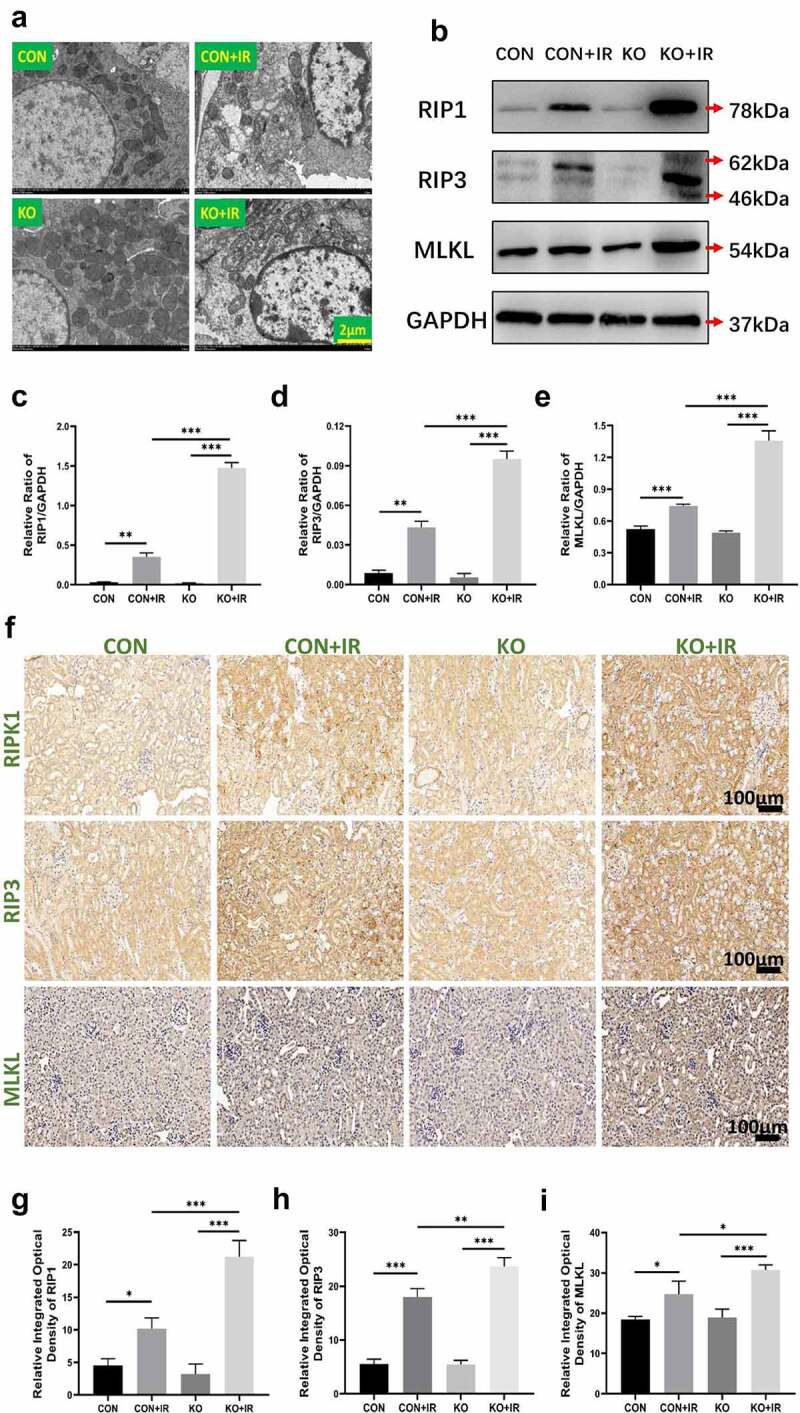
Effect of kidney-specific ALR knockout on necroptosis in kidney tissue in AKI mice. (a) The morphology of renal tissue cells observed by TEM, scale bar:2 µm. Western blotting (b) and quantitative data of the necroptosis-associated proteins RIP1 (c), RIP3 (d) and MLKL (e). The representative images of necroptosis-associated proteins (f) and relative integrated optical density of necroptosis-associated proteins(g-i). Data represent the mean ± standard deviation of at least three independent experiments, *P < 0.05, **P < 0.01, ***P < 0.001.

The genotype identification results of the progeny of ALR^flox/flox^-Ggt1-Cre mice and ALR^flox/flox^ mice after mating are shown in Figure 1. Flox primers and Cre primers were used to identify the genotypes of the mice. If ALR^flox/flox^ was present in the DNA, a 450 bp fragment was amplified; otherwise, two fragments of 450 and 327 were amplified in heterozygotes. The 420 bp fragment was amplified only if the Cre gene was present in the DNA. As shown in Figures 1b, 2, 5, 6, 7, 8, and 9 all showed 420 bp Cre gene PCR results. As shown in Figure 1a, the mice all carried the 450 bp ALR^flox/flox^ gene, on the basis of PCR results. In summary, 2, 5, 6, 7, 8, 9 were ALR^flox/flox^-Ggt1-Cre mice, and 1, 3, 4 were ALR^flox/flox^ mice.

**Figure 3.**
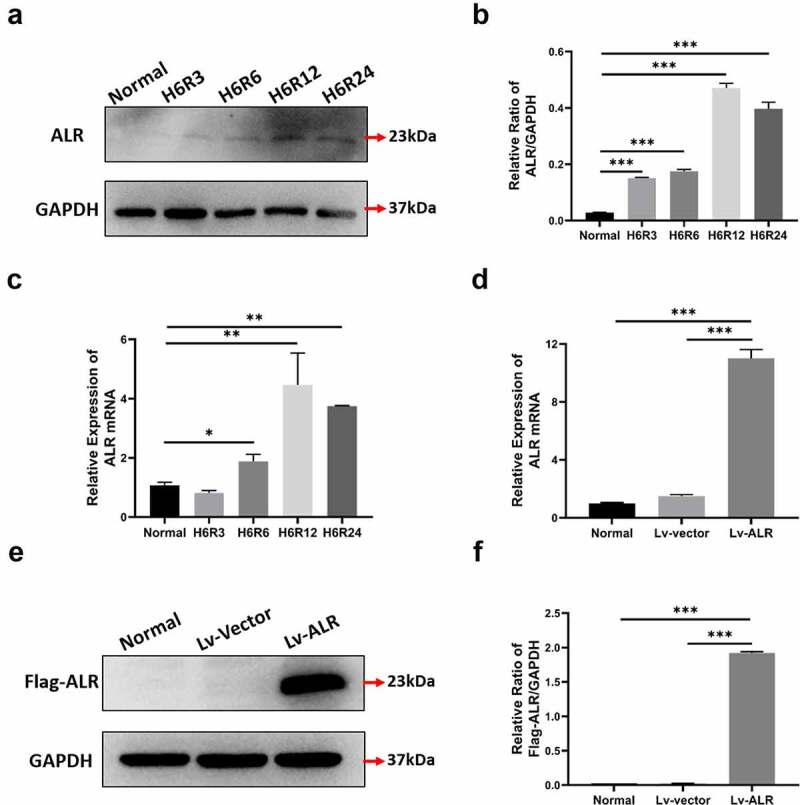
Construction of HK-2 cell line with stable ALR overexpression. The protein and nucleic acid levels of ALR were respectively detected at 3 h, 6 h, 12 h and 24 h during the reoxygenation process after 6 h of hypoxia (a, b) Western blotting and quantitative data on ALR protein expression. (c) RT-PCR analysis of ALR gene expression. (d) RT-PCR analysis of ALR gene expression with anti-Flag antibody. (e, f) Western blotting and quantitative data of ALR protein expression in HK-2 cells with anti-Flag antibody. Data represent the mean ± standard deviation of at least three independent experiments, *P < 0.05, **P < 0.01, ***P < 0.001.

**Figure 4.**
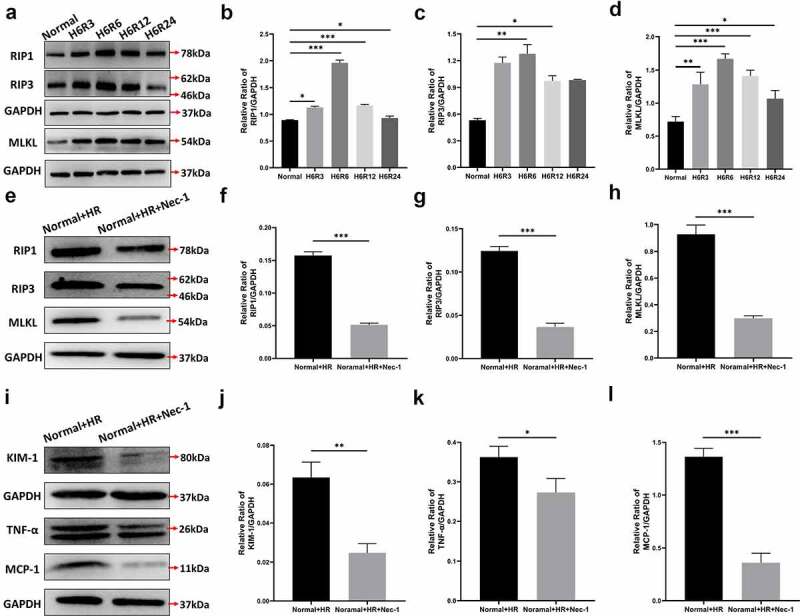
The effect of HR treatment on the necroptosis-associated proteins of HK-2 cells. Western blotting (a) and quantitative data on the necroptosis-associated proteins RIP1 (b), RIP3 (c) and MLKL (d). Western blotting (e) and quantitative data on the necroptosis-associated proteins RIP1 (f), RIPK3 (g) and MLKL (h). Western blotting (i)and quantitative data on KIM-1 (j) and the inflammation-associated cytokines TNF-α (k) and MCP-1 (l). Data represent the mean ± standard deviation of at least three independent experiments, *P < 0.05, **P < 0.01, ***P < 0.001.

**Figure 5.**
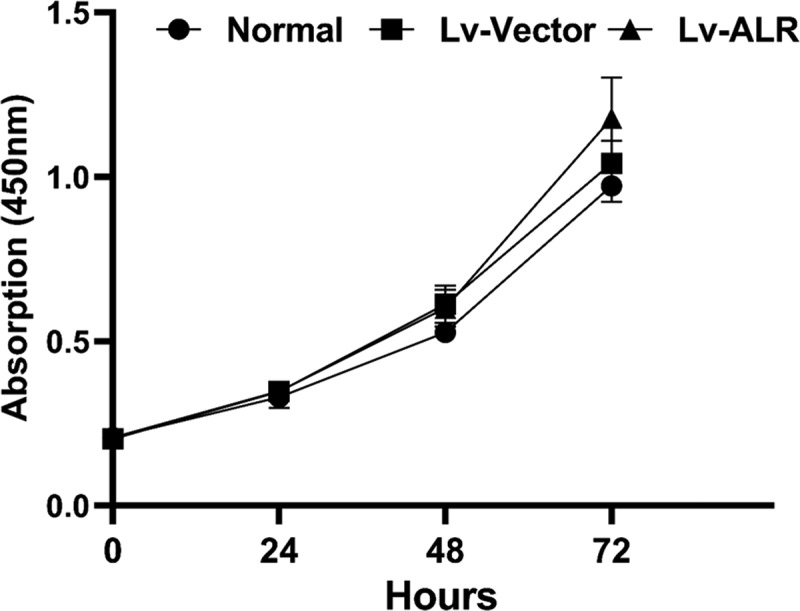
Effect of overexpression of 23-kD ALR on the proliferation of HK-2 cells by CCK-8 analyze. Corresponding absorption at 0, 24, 48, 72 h, in the normal group, negative control group and overexpression group was detected as cell density. Data represent the mean ± standard deviation of at least three independent experiments, *P < 0.05, **P < 0.01, ***P < 0.001.

**Figure 6.**
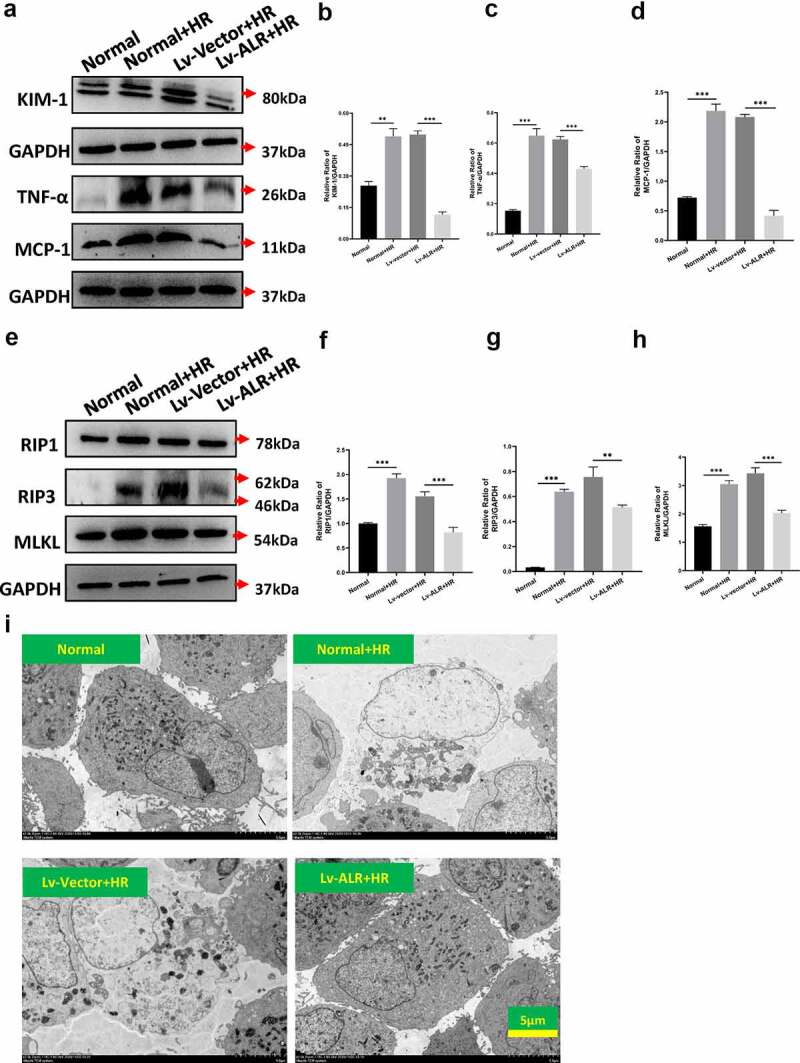
The effects of overexpression of 23-kD ALR on HR-induced HK-2 cell damage and necroptosis-associated proteins. Western blotting (a) and quantitative data on KIM-1 (b) and the inflammation-associated cytokines TNF-α(c) and MCP-1 (d). Western blotting (e) and quantitative data on the necroptosis-associated proteins RIP1 (f), RIP3 (g) and MLKL (h). The effects of overexpression of 23-kD ALR on cell morphology (i) by TEM, scale bar: 5 µm. Data represent the mean ± standard deviation of at least three independent experiments, *P < 0.05, **P < 0.01, ***P < 0.001.

After establishing the ischemia-reperfusion model in the knockout mice, we detected the blood serum creatinine and urea nitrogen concentrations, as shown in Figure 1c,d. In the CON+IR group, compared with the CON group, the serum creatinine and urea nitrogen were significantly higher (P < 0.001). Compared with KO group, the blood creatinine and urea nitrogen levels in the KO+IR group were significantly higher (P < 0.001), thus indicating the AKI mouse model was successfully established. Compared with the levels in the CON+IR group, the blood creatinine and urea nitrogen levels in the kidney ALR-specific knockout mice were significantly higher (P < 0.01), suggesting that the kidney-specific knockout of ALR significantly aggravated the renal function damage in AKI mice caused by IR.

PAS staining and Kidney Injury Score results showed that, compared with the CON group, the CON+IR group exhibited more epithelial cell edema, basement membrane destruction, brush border discontinuity, cell nucleus shedding, renal tubular lumen enlargement, and even tubular and massive infiltration of inflammatory cells (P < 0.001). The damage in the KO+IR group was more evident than the above-mentioned damage in the CON+IR group (P < 0.01), thus suggesting that kidney-specific knockout of ALR significantly aggravated the pathological damage of kidney tissue in AKI mice caused by IR (Figure 1e,f).

### 3.2 The effect of kidney-specific ALR knockout on necroptosis in AKI mice

The death of renal tubular epithelial cells was analyzed by transmission electron microscopy. The results indicated that the renal tubular epithelial cells of mice after acute kidney injury showed signs of ultrastructural necrosis, such as increased volume, intracellular structure disorder, irregular chromatin, swelling of organelles and rupture of the plasma membrane. The ultrastructural characteristics of cell necrosis were more apparent in the KO+IR group (Figure 2a). RIP1, RIP3 and MLKL are key members of the necroptosis pathway, and their expression levels are closely associated with necroptosis. Western blotting (Figure 2b) and statistical analysis (Figure 2c-e) results indicated that the expression of RIP1, RIP3 and MLKL was higher in the CON+IR group than in the CON group (Figure 2c,P < 0.01; Figure 2d, P < 0.01; Figure 2e, P < 0.001), thus confirming that necroptosis in AKI occurred and was induced by IR. Compared with that in the CON+IR group, the expression of RIP1, RIP3 and MLKL in the kidney tissue extracted proteins in the KO+IR group was significantly higher (Figure 2c, P < 0.001; Figure 2d, P < 0.001; Figure 2e, P < 0.001), The IHC images of RIP1, RIP3 and MLKL showed the same trend with Western blot results, and the staining of necroptosis was stronger in IR groups than CON and KO, and the KO+IR mice had stronger staining compared with CON+IR mice (figure 2f) and statistical analysis (Figure 2g-i), thus suggesting that kidney-specific knockout of ALR significantly promotes the necroptosis of kidney tissue in AKI mice induced by IR.

### 3.3 Construction of a cell line with stable overexpression of ALR

To simulate the in vivo IR model in vitro, we established an HK-2 cell IR model with sugar-free serum-free medium combined with three-gas incubator hypoxia-reoxygenation (HR). Western blotting and RT-PCR were used to detect the expression of ALR at different time points after HR treatment. The protein and nucleic acid levels of ALR were detected at 3 h, 6 h, 12 h and 24 h respectively during the reoxygenation process after 6 h of hypoxia. The expression of ALR protein and nucleic acid gradually increased after HR, peaked at H6R12 and then gradually decreased, thus suggesting that ALR is involved in the HR injury process in renal tubular epithelial cells (Figure 3a-c).

Because our previous study indicated that overexpression of ALR had a protective effect on HK-2 cells with AKI, we overexpressed the ALR gene in follow-up experiments. After transfecting HK-2 cells with ALR lentivirus, we used anti-Flag antibody to detect ALR expression levels by real-time PCR and Western blotting. As shown in Figure 3d-f, Lv-ALR was effectively and stably expressed in HK-2 cells after transfection of lentiviral Lv-ALR.

### 3.4 The effect of HR treatment on necroptosis-associated proteins in HK-2 cells

We selected different HR time points to obtain total cell protein and used Western blotting to observe the expression of key proteins of necroptosis, including RIP1 RIP3 and MLKL (Figure 4a). After HR, with increasing reoxygenation time, the expression level of necroptosis-associated protein showed a trend of first increasing and then decreasing (Figure 4b-d). Because the expression at hypoxia (6 h) and reoxygenation (6 h) (H6R6) was highest, we chose H6R6 samples for examination in subsequent experiments.

To further explore the effects of inhibiting necroptosis in AKI, we added the RIP1 inhibitor Nec-1 (50 µm) to the cell culture medium before HR treatment and extracted cellular protein from the H6R6 group. Western blotting was used to detect necroptosis-associated proteins (Figure 4e). Compared with those in the Normal+HR group, the expression levels of RIP1, RIP3 and MLKL in the Normal+HR+Nec-1 group were significantly lower (P < 0.001) (figure 4f-h), thus confirming that Nec-1 significantly inhibits the necroptosis of HK-2 cells. Simultaneously, the expression levels of the inflammatory factors human monocyte chemoattractant protein-1 (MCP-1), tumor necrosis factor-alpha (TNF-α) and the kidney injury factor (KIM-1) in the Normal+HR+Nec-1 group were significantly lower than those in the Normal+HR group (Figure 4J, P < 0.01; Figure 4k, P < 0.05; Figure 4l, P < 0.001). These results again confirmed that necroptosis is important in the pathogenesis of AKI.

### 3.5 CCK-8 detects the effect of overexpression of 23-kDa ALR on the proliferation of HK-2 cells

The effects of overexpressing 23-kDa ALR on cell proliferation were detected by CCK-8 analysis. The proliferation levels of cells in each group were detected at 0, 24, 48 and 72 hours after transfection. After HK-2 cells overexpressed 23-kDa ALR, no significant difference was observed in cell proliferation in the Lv+ALR group compared with the Normal group and the Lv-vector group (P > 0.05; Figure 5).

### 3.6 The effects of overexpression of 23-kDa ALR on HR-induced HK-2 cell damage and necroptosis-associated proteins

Statistical comparison of the protein expression levels of the four experimental groups (Figure 6a) indicated that the expression of KIM-1 (Figure 6b, P < 0.01), TNF-α (Figure 6c, P < 0.001) and MCP-1 (Figure 6d, P < 0.001) in the Normal+HR group was significantly higher, thus suggesting that the in vitro AKI model was successfully established. Compared with that in the Lv-vector+HR group, the expression of KIM-1 (Figure 6b, P < 0.001), TNF-α (Figure 6c, P < 0.001) and MCP-1 (Figure 6d, P < 0.001) in the Lv-ALR+HR group was significantly lower. Our results suggested that hypoxia and reoxygenation cause HK-2 cell damage and increase the expression of inflammatory factors, whereas overexpression of ALR significantly decreases the damage in HK-2 cells and the production of inflammatory factors caused by HR.

After overexpression of ALR, cell protein was extracted from the H6R6 group, and Western blotting was used to detect the level of necroptosis after hypoxia and reoxygenation (Figure 6e). Compared with that in the Normal group, the expression of the necroptotic proteins RIP1 (figure 6f, P < 0.001), RIP3 (Figure 6g, P < 0.01) and MLKL (Figure 6h, P < 0.001) in the Normal+HR group was significantly higher. In the Lv-ALR+HR group, compared with the Lv-vector+HR group, the protein expression of RIP1 (figure 6f, P < 0.001), RIP3 (Figure 6g, P < 0.01) and MLKL (Figure 6h, P < 0.001) was significantly lower, thus confirming that overexpression of ALR significantly inhibited the necroptosis of HK-2 cells caused by HR.

Finally, we observed the morphology of HK-2 cells at the ultrastructural level through TEM. After hypoxia and reoxygenation, HK-2 cells in the Normal+HR group and Lv-vector+HR group showed plasma membrane rupture, increased permeability, cell swelling and decreased organelle content. Compared with those in the Lv-vector+HR group, the cell damage changes in the Lv-ALR+HR group were significantly diminished (Figure 6i). Our findings suggested that overexpression of ALR significantly decreases the pathological damage of HK-2 cells induced by HR.

## 4 Discussion

AKI is a common clinical disease whose incidence has increased in recent years. AKI is a complication in as many as 15% of hospitalizations, and the hospitalization rate of critically ill patients is as high as 50%–60%. Beyond the short-term effects of AKI on patient prognosis, some studies have reported associations between AKI and poor long-term prognosis, such as recurrent AKI attacks in 25%–30% of cases, and rehospitalization rates as high as 40% of patients with cardiovascular events; the risk of chronic kidney disease (CKD) after AKI increases, and the long-term mortality rate increases significantly [31]. However, no effective clinical method is available to prevent or treat AKI.

To date there are three ALR isoforms described, 15-kDa, 21-kDa and 23-kDa, in liver and kidney (and several other organs). Furthermore, the 15-kDa ALR is mainly expressed in the cytosol and was found extracellular, whereas the two longer isoforms, 21-kDa and 23-kDa, where found in the ISM and in the cytosol [32]. However, few studies have examined 21-kDa ALR in the kidney, and its main function remains unclear. Many studies have been conducted on 15-kDa and 23-kDa ALR in the kidney. Our previous research results have indicated that 15-kDa and 23-kDa ALR protects organisms against IR-induced AKI by inhibiting renal tubular epithelial cell apoptosis, regulating autophagy and decreasing renal tubular epithelial cells [6,25,27]. To further explore the protective effect of ALR against AKI, we constructed kidney-specific ALR gene knockout SPF grade C57mice. The CON+IR group showed clear renal function and pathological damage, in contrast to the CON group, thus confirming successful construction of the IR-induced AKI model. Simultaneously, the renal function damage and pathological damage were clearer in mice with specific knockout of ALR in the kidney. In agreement with the findings from our previous study, ALR has a protective effect in IR-induced AKI model mice.

The mechanisms underlying cell death have long been a focus of life science research. Necroptosis is an important form of programmed necrosis discovered in recent years, whose importance has been confirmed in many diseases. Necroptosis has several notable characteristics: (1) It has the morphological characteristics of necrotic cell death, such as loss of cell plasma membrane integrity and loss of mitochondrial membrane potential. (2) Autophagy is a common downstream response to necroptosis, which is often accompanied by autophagosome formation. (3) Inflammatory cell infiltration is more common in tissues that undergo necroptosis, and some necrosis-like cells produce large amounts of reactive oxygen free radicals. (4) Necroptosis is specifically inhibited by the small molecule Nec-1 [33,34]. Liu et al [35]. have reported necroptosis in AKI induced by IR. Consistently, Shen [36] et al. have also observed the up-regulation of necroptosis-associated proteins in an ischemic AKI model and have reported that Nec-1 pretreatment has a renal protective role in IR kidney injury. These studies suggest that necroptosis is a key mechanism in the pathogenesis of AKI.

Apoptosis, necroptosis and autophagy have been found to be associated. Our previous studies have confirmed that ALR inhibits apoptosis in AKI and regulates autophagy. Consequently, we assumed that ALR might regulate necroptosis in AKI. In this study, we observed typical necrotic morphological changes after IR injury in mice. We conducted Western blotting to detect the changes in necroptotic protein after IR injury in mice. Compared with that in the CON group, the expression of RIP1, RIP3 and MLKL was significantly higher in the CON+IR group, thereby confirming that IR induced necroptosis in AKI. Compared with that in the CON+IR group, the expression of necrosis-related proteins in the KO+IR group was also significantly higher (Figure 2c-e). Therefore, our results demonstrated that kidney-specific ALR knockout further aggravates necroptosis.

We observed the changes in ALR after hypoxia and reoxygenation. In different periods of hypoxia and reoxygenation, ALR gradually increased and then decreased, and peaked at H6R12; the expression remained high at H6R24 (Figure 3). In agreement with previous research findings, ALR compensatively increases after renal IR injury, thus exerting a protective effect on the kidney [37]. The protective mechanism of ALR against renal injury must be further explored.

Through our in vitro experiments, we found that necroptosis occurred in HK-2 cells after hypoxia and reoxygenation. In different periods of hypoxia and reoxygenation, RIP1, RIP3 and MLKL showed a trend of increasing first and then decreasing, and their levels peaked at H6R6 (Figure 4a-d). Consequently, we chose H6R6 as the time point for subsequent experiments.

We transfected 23-kDa ALR lentivirus into HK-2 cells and selected stable 23-kDa ALR overexpressing cells by using purine. Cell viability was detected with a CCK-8 kit. Overexpression of the 23-kD ALR scarcely influenced cell viability. Therefore, we speculate that the function of ALR against IR-induced AKI does not play a role in promoting cell proliferation, thus excluding the effect of proliferation on the experimental results.

Nec-1 has been found to specifically inhibit the activity of RIP1 and subsequently block the formation of necrosomes. It is currently the most widely studied inhibitor of necroptosis. To confirm that the inhibition of necroptosis protects against renal damage in AKI caused by HR, we added Nec-1 before hypoxia and reoxygenation treatment in HK-2 cell culture. The results of Western blotting indicated that after addition of Nec-1, the expression of RIP1, RIP3 and MLKL significantly decreased, thus confirming that Nec-1 specifically inhibits RIP1 and necroptosis. The protein expression of the kidney injury molecule KIM-1 and the inflammatory factors TNF-α and MCP-1 revealed that inhibition of necroptosis decreased HR-induced kidney injury, whereas inhibition of necroptosis inhibited the inflammation caused by AKI. Necroptosis is programmed cell death induced by various stimuli both inside and outside cells, including TNF family members, Fas ligand, interferon and oxidative stress. TNF-α-induced necroptosis is the most common form [38]. Recent studies have demonstrated that necroptosis triggers a severe inflammatory response by destroying cell membranes and promotes the release of endogenous pro-inflammatory molecules called damage-associated molecular patterns, thus causing severe inflammatory reactions and more excessive necrosis. The formation of a positive feedback loop promotes further kidney damage. Fibrosis, tissue damage and organ failure would occur if this self-amplified inflammatory circuit were not inhibited [39,40]. Therefore, specific inhibition of necroptosis may decrease renal tubular cell damage and inflammation.

To further illustrate the regulatory effect of ALR on necroptosis, we observed the changes in the morphology of HK-2 cells after hypoxia and reoxygenation through TEM. After hypoxia and reoxygenation, HK-2 cells experienced plasma membrane rupture, increased permeability, cell swelling and decreased organelle content, findings consistent with the morphological characteristics of necrosis. Simultaneously, we detected the changes in necroptosis-associated proteins. Western blotting indicated that the expression of RIP1, RIP3 and MLKL in the Lv-ALR+HR group was significantly lower than that in the Lv-vector+HR group. In agreement with the results of *in vivo* experiments, our findings indicated that ALR regulates necroptosis. However, whether ALR directly targets necroptosis-mediated cell death or indirectly regulates the RIP1/RIP3/MLKL pathway is unclear. Therefore, the basic mechanism must be further clarified in future research. The cell death pathway of AKI after renal IR injury is complex and interrelated. The available evidence has shown that multiple cell death pathways including apoptosis, necrosis and ferroptosis are involved in this process. Meaningful protection may require targeting multiple pathways, because inhibiting one pathway might make another pathway sensitive [41]. The most effective strategy to limit the severity of AKI may be a combination therapy that antagonizes these approaches. Therefore, the effect of ALR on necroptosis is also important.

Our research revealed that ALR prevents and ameliorates AKI induced by hypoxia and reoxygenation through the inhibition of RIP1/RIP3/MLKL-mediated necroptosis. Our findings highlight the preventive and therapeutic potential of ALR in the treatment of AKI.

## 5 Conclusion

In summary, our results demonstrate for the first time that kidney-specific knockout of ALR aggravates IR-induced acute kidney injury, and that ALR can ameliorate IR-induced acute kidney injury by regulating necroptosis through the RIP1/RIP3/MLKL pathway. This study provides a theoretical basis for the prevention and treatment of AKI caused by IR.

## Highlights

- Kidney-specific knockout of ALR exacerbates acute kidney injury.
- The expression of RIP1/RIP3/MLKL increased in acute kidney injury induced by IR.
- ALR regulates necroptosis through the RIP1/RIP3/MLKL pathway.
